# *Effects of ethanol extract of *Salvia hydrangea* on hepatic and renal functions of streptozotocin-induced diabetic rats*

**Published:** 2015

**Authors:** Ali Zarei, Gholamhassan Vaezi, Ali Akbar Malekirad, Mohammad Abdollahi

**Affiliations:** 1 *Department of Biology, Damghan Branch, Islamic Azad University, Semnan, Iran*; 2 *Department of Biology, Payame Noor University,Tehran, Iran*; 3 *Faculty of Pharmacy and Pharmaceutical Sciences Research Center, Tehran University of Medical Sciences, Tehran, Iran*

**Keywords:** *Diabetes*, *Liver*, *Kidney*, *Streptozotocin*, *Glibenclamide*, *Salvia hydrangea*

## Abstract

**Objectives:**

A wide range of liver and kidney disorders are associated with diabetes and there is a mutual relationship between diabetes and these diseases. Herbal medicine with having abundant ingredients is one of these options. The goal of this study was to compare the effects of alcoholic extract of aerial parts of *Salvia hydrangea* with glibenclamide on functional tests of liver and kidney in diabetic rats induced by streptozotocin.

**Materials and Methods:**

In this study, 35 male Wistar rats were divided into five groups (n= 7 in each group): control, diabetic control, and three experimental diabetic groups. The controls had normal access to water and food, the diabetic control group was given drug solvent and the three experimental groups received ethanol extract of *Salvia hydrangea* at doses of 100 and 200 mg and glibenclamideat a dose of 10 mg/kg/BW by gavage, respectively. To induce diabetes, a single dose of streptozotocin (60 mg/kg/BW) was injected to rats intraperitoneally. Blood samples were collected at day 21 from all groups and the related blood factors were measured and analyzed.

**Results:**

The results showed that the levels of creatinine, urea, aspartate aminotransferase (AST), and fasting blood sugar (FBS) in all diabetic groups increased compared to the control group. In all experimental groups and the group which received glibenclamide,a significant decrease was shown compared to the diabetic group (*p<0.05*).

**Conclusion:**

The consumption of alcoholic extract of aerial parts of *Salvia hydrangea *may have beneficial effects on the reduction of diabetic complications by lowering blood sugar without any adverse effects on the kidney and liver tissue.

## Introduction

The first complete clinical description of diabetes was given by the ancient Greek physician Aretaeus of Cappadocia (1^st^ century CE), who also noted the excessive amount of urine which passed through the kidneys (Leonid Poretsky2009 ▶). Aretaeus attempted to treat it but he could not give a good prognosis. He commented that "life (with diabetes) is short, disgusting, and painful" (Medvei and Victor, 1993 ▶). In medieval Persia, Avicenna (980–1037) provided a detailed account on diabetes mellitus in The Canon of Medicine, "describing" the abnormal appetite and the collapse of sexual functions," and he also documented the sweet taste of diabetic urine. Similar to Aretaeus, Avicenna recognized the primary and secondary diabetes (Ahmed, 2002 ▶). 

As predicted by the World Health Organization, the number of people suffering from this disease will amount to 370 million by 2030 (Ozougwu et al., 2013 ▶). There are two main types of diabetes mellitus: diabetes mellitus type I, which is also called insulin-dependent and is caused by impairment of insulin secretion from pancreatic cells. The second one is diabetes type II, also called non-insulin dependent diabetes, which is caused by a decreased sensitivity to insulin target tissues (Ozougwu et al., 2013 ▶;Yi Lin and Zhongjie, 2010 ▶). In both types of diabetes, all food metabolisms change. The effects of insulin reduction or insulin resistance on glucose metabolism prevent proper absorption and utilization of glucose and thus its level increases in blood (Guyton and Hall, 2012 ▶).

A wide range of kidney and liver diseases, such as diabetic nephropathy, abnormally elevated liver enzymes, non-alcoholic fatty liver disease, cirrhosis, and chronic liver dysfunction are associated with diabetes (Lee et al., 2014;Leeds et al., 2009 ▶). Studies show that there is a mutual relationship between diabetes, liver disease, vascular disorders, and renal damage (SheybaniAsl et al., 2014 ▶;Ghamarian et al., 2012 ▶).

Today, due to the side effects of chemical drugs, drug tolerance, rise of the complications of the disease -despite careful management-, and high expenses imposed on society and individuals, the use of medicinal herbs and traditional medicine has become a priority. This approach has aimed at treating patients in a faster, cheaper, and more relaxed way with fewer complications. Previous studies have shown that a wide range of medicinal plants with active ingredients such as alkaloids, peptidoglycans, terpenoids, amino acids, and inorganic ions are effective in treating diabetes. Thus, researching to identify these active ingredients and to determine their degree of efficacy in reducing the adverse effects of the disease in various tissues including liver and kidney is of utmost value and importance (Asgary et al 2010 ▶, Kazemi et al 2010 ▶;Mokhtary et al., 2013 ▶). 

Among the most popular herbs used in traditional medicine to treat diabetes one can refer to many different species of salvia. Natural products which possessanti diabetic potentials exert their effects through their insulin mimetic properties, insulin secretion, intestinal glucose transporter inhibition, or insulin-dependent metabolic processes (Feldman, 1988 ▶).Salvia genera are commonly called sages which are the largest members of the family of Lamiaceae. In Iran, there are about 58 species in the genus among which 17 species including hydrangia are native (Pen and Fang, 2003 ▶). Salvia species are also rich sources of polyphenolic flavonoids and phenolic acids (Chhetri et al., 2005 ▶;Ghosal et al., 1980 ▶). Many species and compounds isolated from them have significant antioxidant properties which are exerted through enzymatic and non-enzymatic pathways (Chhetri et al., 2005 ▶;Kawanishi et al., 2003 ▶). 

Alpha-amylase is one of the key human enzymes which is responsible for the breakdown of starch into simpler sugars. The inhibition of this enzyme can inhibit carbohydrate digestion and decrease glucose absorption. The activity of this enzyme is also reduced by various species of salvia including hydrangea (Nickavar et al., 2008 ▶). Three isopenoids (salvadonin, proves kone, and hydrangenone) have been isolated from salvia species. This plant with the scientific name of *Salvia hydrangea *is commonly called Gol-e-arooneh in Fars province of Iran (Figure 1) (Moridi et al., 2012 ▶).

According to studies,terpenoidswereuseful in the treatment of diabetes (Upenderaro et al., 2001 ▶). There are a number of active ingredients in the plant that are potentially able to inhibit the uptake of glucose and have antioxidant and antidiabetic properties. Therefore, the present research was carried out to study the effects of the alcoholic extract of *Salvia hydrangea* on controlling hyperglycemia as well as on liver and kidney disorders in streptozotocin-induced diabetes in rats.

## Materials and Methods

**Study design**

The present study was an experimental study one on 35 male Wistar rats purchased from the center for breeding laboratory animals in Arak Medical University (Arak, Iran). The rats were divided into five groups (n=7 in each group): 1) a control group that received normal diet and water, 2) the diabetic controls which received streptozotocin (STZ) at a single dose of 60mg/kg/BW intraperitoneally (i.p.), 3 and 4) diabetic groups treated with alcoholic extract of *Salvia hydrangea* at the dose of 100 and 200mg/kg/BW, respectively, and 5) diabetic group treated with glibenclamide (Kimidaru company, Iran) at the dose of 10mg/kg/BW viagavage. During the test, animals were kept in standard conditions of light, temperature, and humidity and the principles of working with laboratory animals approved by the Ministry of Health and Medical Education were entirely observed (Anand and Murali, 2007 ▶).

**Method of animal diabetization**

STZ was purchased from Upjohn Company, USA. The animals were kept hungry with free access to water for twelve hours before injection. To diabetize animals, a single dose of STZ was injected i.p.After 48 hours, fasting blood glucose levels were measured with Easy Gluco (Combo 142, USA) to ensure diabetes. Blood sugar levels above 220 mg/dl were considered as the basis for diabetes diagnosis. Diabetic rats showed symptoms of frequent urination and polydipsia. Then, the diabetized animals received daily doses of the extract or glibenclamide by gavage over a period of three weeks (Thiruvenkatasubramaniam et al., 2010 ▶, Zar et al., 2012 ▶).

All groups were kept fasting over the night prior to blood sampling while they had free access to water. In the first day of the experiment and before the diabetization of the rats (as day zero) their blood sugar levels were measured and recorded and since then these measurements were repeatedand recorded on a weekly basis (**Nan et al., 2001**). Experimental period lasted for 21 days and the rats were gavaged daily at 9 am.

**Biochemical assays**

After applying ether for mild anesthesia, blood samples were collected from heart and after centrifugation (Mini Spin Eppendorf, Germany) at the rate of 3000 rpm, the isolated sera were transferred to the laboratory for measurement of blood glucose, alanine amino transferase (ALT), aspartate amino transferase (AST), alkaline phosphatase (ALP), albumin, urea, and creatinine. To measure liverenzymes, radio immunoassay method (RIA), Pars Azmoon kit (Iran) and autoanalyzer (RA 1000,Technicon Instruments, USA)were used. 

**Extraction method**

In spring, *Salvia hydrangea *species were collected from Fars (Iran). The genus and species were identified by Sonboli and colleagues and kept at the Institute of Medicinal Plants in Shahid Beheshti University with the herbarium code of MPH-761 (Sonboliet al., 2005 ▶). To prepare the plant extract, after collecting the aerial parts and removing impurities, 600 g of the plant was ground and mixed with ethyl alcohol 90% by the ratio of 1 to 5. After 24 hours, the mixture was placed in a shaker. Then, the extract was filtrated, and ethyl alcohol 70% was poured on the remaining raff. This mixture was again placed on the shaker for 24 hoursandthe obtained extract was filtrated and then added to the first one.Later, the whole extract was distilled in the vacuum distillation unit at 60°C and at the rotation rate of 70% until the remaining volume was one-fifth of the initial one. The tank was then removed and after cooling down, the extract was decanted for three times, each time with 50cc of chloroform. The remainder was poured into Petri and dried in an Avon (Finetech, Korea) at 50°C. The obtained extract (about 10 g per 100 g of crushed plant) was mixed with normal saline to obtain different concentrations of it in terms of mg/Kg/BW (Zarei et al., 2013a ▶).

**Data analysis**

The values of parameters were compared between groups by oneway ANOVA followed by Duncan’s post-hoc test. All the data analyses were performed using SPSS 17 software. Data are presented as mean±SD and significance was taken at *p*<0.05.

## Results

In this experiment, after two days, fasting blood sugar (FBS) levels in the diabetic groups significantly increased compared to the control group (Table 1). Results also showed that after the administration of salvia extract, FBS levels significantly decreased in experimental groups 1 and 2 which received doses of 100 and 200 mg/kg/BW as well as in the glibenclamide group compared to diabetic control group after three weeks (*p<0.05*).

In addition, at the end of the second and third weeks, in groups which received doses of 100 mg/kg and 200 mg/kg of the extract, no significant changes in blood sugar were seen in comparison to glibenclamide group or to each other (Table 1).

 The results of the statistical analysis (Table 2) showed that creatinine levels in diabetic control group compared to the control group had a significant increase; and its level decreased significantly in all treatments groups compared to diabetic control group.

Urea levels in diabetic control group compared to the control group showed a significant increase. Its rate decreased significantly in all treatments groups compared to diabetic control group.In addition, urea changes amongall of the experimental groups and compared to each other were not significant. Plasma albumin in diabetic control group compared to the control group did not show any significant changes. In addition, the rate of albumin change was not statistically significant in any of the experimental groups compared to diabetic control group and to each other. ALP levels significantly increased in diabetic control group, but its level in the experimental groups showed no significant changes compared to the diabetic control group.There were no significant changes between experimental groups, either.

**Table 1:** Comparison of fasting blood sugar (FBS) in different groups using different doses of ethanol extract of *Salvia hydrangea *and glibenclamide.

**FBS**	**Control**	**Sham** **(diabetic** **contro)**	***Salvia hydrangea *** **(100mg/kg)**	***Salvia hydrangea *** **(200mg/kg)**	**Glibenclamide** **(10mg/kg)**
**Pre-** **diabetes**	70.5±15.3	70.2±12.7	70±14.8	69.7±17.5	69±16.7
**48 hours after diabetization**	70.5±13.6	302.7±71.1	385.8±115.3	354.2±129.3	302.9±1.1
**End of first week**	72.7±14.6	339.3±72.8	231.7±116.2†	326.2±133.5	302.4±76.8†
**End of second week**	68.8±19.2	352±80.3	109.8±74.7	166.3±143.2	132.6±89.4
**End of third week**	64.7±17	353.3±123.4*	103.8±21	163.2±95.1	161.1±84.3

* Marks a significant level compared to the control group.

† Marks a significant level compared to the diabetic control group.

**Table2:** Comparison of the renal and liver functional test in different groups using different doses of ethanol extract of *Salvia hydrangea *and comparing them with glibenclamide.

**Parameters**	**Control**	**Diabetic control**	***Salvia hydrangea *** **(100mg/kg)**	***Salvia hydrangea *** **(200mg/kg)**	**Glibenclamide (10mg/kg)**
**Creatinine(mg/dl)**	0.427±0.02	0.575±0.02	0.455±0.01	0.428±0.02	0.402±0.15
**Urea(mg/dl)**	30.75±2.5	145.12±29.4	81.12±14	59.16±8.24	84.32±0.15
**Albumin(g/dl)**	3.41±0.11	3.72±0.02	3.55±0.1	3.31±0.2	3.32±0.2
**ALP(U/l)**	419±33	975±116	1015±255	1258±217	1422±145
**AST** **(U/l)**	140±5.1	166±4.9	165±12	134±12 † α	101±6.9
**ALT** **(U/l)**	52.92±0.4	72.80±5.8	71.32±4.13	75.86±7.04	73.17±1.08

* Marks a significant level of diabetic control group compared to the control group.

† Marks a significant level of experimental groups compared to the diabetic control group.

α  Marks a significant level of the group receiving the extract as compared to glibenclamide group

AST levels significantly increased in diabetic control group compared to the control group. However, its level in the experimental group which received the higher dose of the extract (200mg/kg/BW) and the experimental group with glibenclamide (10mg/kg/BW) decreased significantly compared to the diabetic control group. AST levels in the group receiving glibenclamide compared to the groups receiving the extract showed a significant decrease (*p=0.003*). 

ALT levels significantly increased in diabetic control group compared to the control group. However, these amounts were not significant in any of the experimental groups compared to the diabetic control group. There were no significant changes among all of the experimental groups, either.

## Discussion

The results of the present study indicated that i.p. injection of STZ destroyed beta cells and thus induced diabetes type I in rats in which glucose level significantly increased compared to controls. However, blood glucose levels reduced in all experimental groups which received saliva extract and glibenclamide. Streptozotocin is a diabetogenetic, hepatotoxic, and nephrotoxic substance which by damaging the pancreatic beta cell membrane, fragmenting DNA, and reacting with enzymes such asglucokinase, sharply decreases insulin levels and thereby increases glucose levels in animals (Perfumi et al., 1991 ▶).

STZ increases mRNA expression of the liver glucose-6-phosphate dehydrogenase enzyme and thereby causes blood glucose to increase. Studies have also shown that diabetes could cause dyslipidemia and fatty liver. The reason for this is either the increased flow of fatty acids to the liver as the result of insulin reduction or decreased lipoprotein secretion from the liver due to a shortage of apolipoprotein B synthesis (Mehrabani and Heidary, 2010).

When the antidiabetic properties of *Salvia hydrangea* are compared to other species of salvia, whose antidiabetic properties are determined in many studies, it can be concluded that the plants studied here had better effects than *Salvia lavandulifolia* and *Salvia fruticosa*. (Zarzalo et al., 1990 ▶;Perfumi et al., 1991 ▶).*Salvia lavandulifolia *lowered blood sugar in diabetic rabbits, whereas in normal animals it prevented hyperglycemia only by affecting the intestinal absorption of glucose. Therefore, it is said to have an insulin dependent effect (Mehrabani and Heidary, 2010).

Results of studies done by Perfumi and colleagues demonstrated that the administration of a single dose of 250 mg/kg of Salvia *lavandulifolia* leaf extract as gavage to normoglycemic rabbits and also to the normoglycemic rabbits marked with alloxan administration, could only decrease blood glucose levels in hyperglycemic rabbits. While the administration of a single oral dose of the extract to rats previously fed with glucose resulted in reduced blood glucose in both diabetic and control groups. Therefore, they concluded that the hypoglycemic effects are mainly due to decreased intestinal absorption of glucose (Perfumi et al., 1991 ▶).

*Salvia hydrangea *appeared to reduce blood glucose levels and intestinal glucose absorption in diabetic rats with no changes in plasma insulin levels (Eidi et al., 2005 ▶).

Glibenclamide, also known as glyburide, is an antidiabetic drug in a class of medications known as sulfonylureas. Long-term use of glibenclamide increases the sensitivity of peripheral tissues such as liver, muscles, and adipose tissues to insulin, but this is a minor mechanism (Serrano-Martin et al., 2006 ▶). As it was shown in this study, although this drug lowered blood sugar, it had no significant effect on liver enzyme levels and in some cases it even increased the levels of these enzymes. Plants used in the treatment of diabetes often exert their effects through increasing insulin secretion, increasing glucose reabsorption by skeletal muscle and adipose tissues, inhibiting intestinal absorption of glucose, and inhibiting hepatic glucose production. The main active components for diabetes are: alkaloids, glycosides, steroids, carbohydrates, glycopeptide, terpenoids, amino acids, and inorganic ions (Eidi et al., 2005 ▶;Maroo et al., 2002 ▶).

Salvia species are also rich sources of phenolic acids and flavonoids and quercetin (Chhetri et al., 2005 ▶;Ghosal et al., 1980 ▶). Flavonoids especially have beneficial effects on diabetes. They do so by exerting an inhibitory effect on aldose reductase enzyme which may play a role in diabetes complications (Lim et al., 2006 ▶). In another study, quercetin decreased glucose and increased plasma insulin levels in streptozotocin-induced diabetic rats (Vessal et al., 2003 ▶). Many species of this genus as well as their isolated compounds showed significant antioxidant properties which are exerted via enzymatic and non enzymatic pathways (Chhetri et al., 2005 ▶;Kawanishi et al., 2003 ▶). Alpha-amylase is one of the key human enzymes responsible for the breakdown of starch into simpler sugars. Inhibition of this enzyme can inhibit carbohydrate digestion and reduce glucose absorption. Activity of this enzyme is also reduced by various species of salvia including hydrangea (Nickavar et al., 2008 ▶).

The increase in the activity of AST, ALT, and ALP enzymes in plasma may be caused by leakage of these enzymes from the liver cells into the bloodstream, which in turn causes hepatotoxic effects of streptozotocin (Navarro et al., 1993 ▶). Administration of the alcoholic extract of Salvia hydrangea decreased AST, ALT serum levels which may be related to a decrease in ATP production in the absence or shortage of insulin. This may be related to decreased levels of plasma proteins in diabetic rats.Moreover, in diabetes, increased protein catabolism may cause direct damage to the synthesis and secretion of albumin and thus may result in decreased albumin level (Chandramohan et al., 2009 ▶) .In addition, considering the antioxidant properties of the plant, a reduction in the activity of liver enzymes in the experimental groups which received *Salvia hydrangea* extract was expected. However, the extract only reduced the amount of AST and was ineffective on ALT and ALP.

The study done by Giacco et al. (2010) suggested that hyperglycemia increased the advanced glycation end products (AGE), facilitated the production of free radicals, and reactive oxygen species (ROS) which were produced by malfunctioning endogenous scavengers such as superoxide dismutase (SOD) and catalase (Giacco and Brownlee, 2010). Oxidative stress induced by superoxide anions are involved in the pathophysiology of diabetic nephropathy (Giacco and Brownlee, 2010;Kaneto et al., 2007 ▶). Alcoholic extract of *Salvia hydrangea* protected the kidney in experimental diabetes which, in addition to hypoglycemic effects, may be attributed to antioxidant and oxidative stress reducing properties of the compounds in the extracts (Amouoghli-Tabrizi et al., 2011 ▶).

*Salvia hydrangea* has potent antioxidant properties. It also contains phenolic compounds (Chhetri et al., 2005 ▶; Kawanishi et al., 2003 ▶). Polyphenolic compounds and flavonoids can also revive the cells against glutathione depletion and protect them by increasing the capacity of anti-oxidant enzymes (glutathione, glutathione reductase, glutathione peroxidase, and catalase) (Zarei et al., 2014b ▶).

Another research also demonstrated that enzymatic and non enzymaticdefense systems scavenging free radicals weaken in diabetic cells, whereas lipid peroxidation increases in cells (Sellamuthu et al., 2013). Accordingly, multiple injuries and severe damages occur in different organs in such a way that liver and kidney failures become a major cause of death in diabetic patients (Pickup and William, 1997 ▶).

Previous research showed that the intake of antioxidants in diabetic rats reduced renal damage (Thomson et al., 2007 ▶; Zar et al., 2012 ▶). In this study, consumption of alcoholic extract of *Salvia hydrangea* in the treated diabetic groups reduced serum levels of creatinine and urea nitrogen compared to the diabetic control group. It appears that the extract of *Salvia hydrangea*, strengthened the antioxidant system in rats and increased their ability to cope with oxidative stress and thus the subsequent renal damage reduced. Urea and creatinine levels rose by enhancing the function of the damaged kidneys in diabetic rats. These results were consistent with those of previous studies done by Mandade and colleagues on aqueous/alcoholic extract of *Hibiscus rosasinesis* (Mandade and Sreenivas, 2011 ▶).

The findings showed that long-term administration of *Salvia hydrangea* extract was able to lower the glucose level which had increased following the administration of streptozotocin in diabetic rats. Moreover, some flavonoids in the extract of *Salvia hydrangea* reduced liver and kidney damage, followed by decreased liver enzymes and renal factors which arein agreement with the results of arecent study(Yakubu MT and Musa IF, 2012 ▶).

Many plants can inhibit lipid peroxidation because they contain a plenty of antioxidants. This property is applied when the existing oxidizing structure is broken down by cytochrome P_450_ and free radical neutralization (Zarei et al., 2014c ▶). *Salvia hydrangea*may inhibit oxidative stress inducted by streptozotocin in rats (Chhetri et al., 2005 ▶; Kawanishi et al., 2003 ▶). 

In present study (Table 2),from the changes in the concentrations of creatinine and urea, as well as in liver enzymes, it can be concluded that *salvia* extract at the doses studied did not have any harmful effects on kidney and liver function, which is a remarkable privilege for new investigational drugs. Moreover, the pattern of changes in different groups, did not show significant differences between doses of 100 and 200 mg/kg of the extract. In other words, the changes observed in this study, at least in the doses mentioned, are not dose-dependent.

The present study showed that diabetes caused significant functional impairments in liver and kidney. These impairments were greatly alleviated or cured in diabetic rats treated with Salvia hydrangeaor glibenclamide.
